# Machine learning techniques for personalized breast cancer risk prediction: comparison with the BCRAT and BOADICEA models

**DOI:** 10.1186/s13058-019-1158-4

**Published:** 2019-06-20

**Authors:** Chang Ming, Valeria Viassolo, Nicole Probst-Hensch, Pierre O. Chappuis, Ivo D. Dinov, Maria C. Katapodi

**Affiliations:** 1 Nursing Science, Faculty of Medicine, University of Basel, Bernoullistrasse 28, Room 118, 4056 Basel, Switzerland; 2 Oncogenetics and Cancer Prevention, Geneva University Hospitals, Geneva, Switzerland; 3 Swiss Tropical and Public Health Institute, University of Basel, Basel, Switzerland; 4 Genetic Medicine, Geneva University Hospitals, Geneva, Switzerland; 5 Department of Computational Medicine and Bioinformatics, University of Michigan, Ann Arbor, MI USA; 6 Michigan Institute for Data Science, University of Michigan, Ann Arbor, MI USA; 7 Statistics Online Computational resource, University of Michigan, Ann Arbor, MI USA; 8 University of Michigan School of Nursing, Ann Arbor, MI USA

**Keywords:** Breast cancer, Risk prediction, Machine learning, Big data, Personalized medicine, Cancer screening

## Abstract

**Background:**

Comprehensive breast cancer risk prediction models enable identifying and targeting women at high-risk, while reducing interventions in those at low-risk. Breast cancer risk prediction models used in clinical practice have low discriminatory accuracy (0.53–0.64). Machine learning (ML) offers an alternative approach to standard prediction modeling that may address current limitations and improve accuracy of those tools. The purpose of this study was to compare the discriminatory accuracy of ML-based estimates against a pair of established methods—the Breast Cancer Risk Assessment Tool (BCRAT) and Breast and Ovarian Analysis of Disease Incidence and Carrier Estimation Algorithm (BOADICEA) models.

**Methods:**

We quantified and compared the performance of eight different ML methods to the performance of BCRAT and BOADICEA using eight simulated datasets and two retrospective samples: a random population-based sample of U.S. breast cancer patients and their cancer-free female relatives (*N* = 1143), and a clinical sample of Swiss breast cancer patients and cancer-free women seeking genetic evaluation and/or testing (*N* = 2481).

**Results:**

Predictive accuracy (AU-ROC curve) reached 88.28% using ML-Adaptive Boosting and 88.89% using ML-random forest versus 62.40% with BCRAT for the U.S. population-based sample. Predictive accuracy reached 90.17% using ML-adaptive boosting and 89.32% using ML-Markov chain Monte Carlo generalized linear mixed model versus 59.31% with BOADICEA for the Swiss clinic-based sample.

**Conclusions:**

There was a striking improvement in the accuracy of classification of women with and without breast cancer achieved with ML algorithms compared to the state-of-the-art model-based approaches. High-accuracy prediction techniques are important in personalized medicine because they facilitate stratification of prevention strategies and individualized clinical management.

## Background

Since 2009, the U.S. Preventive Services Task Force recommends breast cancer screening with biannual mammograms for women age 50 to 74 years old [1]. In 2013, Switzerland also adopted a national strategy, recommending biannual breast cancer screening for women over 50 [2, 3]. Age over 50 years is the sole risk factor considered for entering a population screening program [4–6]. However, about 25% of breast cancer patients are diagnosed in women under 50 years old [7, 8]. Mammograms are less effective as a breast cancer screening tool for younger women, who are more likely to have dense breast tissue, compromising the utility of routine mammograms in this age group. This contributes to diagnostic delays and increased morbidity and mortality [8, 9]. Risk-based screening could be more effective, less morbid, and more cost-effective [10–17]. Comprehensive breast cancer risk prediction models, able to classify women into clinically meaningful risk groups, will enable identifying and targeting women at high-risk, while reducing interventions in those at low risk.

The Breast Cancer Risk Assessment Tool (BCRAT), also known as the Gail model, and the Breast and Ovarian Analysis of Disease Incidence and Carrier Estimation Algorithm (BOADICEA) model were developed to identify high-risk women based on known risk factors and have been integrated into clinical guidelines to help guide decision making about breast cancer risk management [18, 19]. BCRAT was developed and validated with data from the US Surveillance, Epidemiology, and End Results registry [20]. The model uses eight risk factors, i.e., age, age of menarche, age of first live birth, number of previous biopsies, benign disease, *BRCA* mutations, race, and number of first-degree relatives affected with breast cancer, to calculate 5-year and lifetime risk for women older than 35 years old [21]. The National Comprehensive Cancer Network suggests using BCRAT to identify women with a 5-year risk greater than 1.66% and women with remaining lifetime risk greater than 20%, who could consider risk-reducing chemo-prevention and annual screening with mammograms and MRIs (magnetic resonance imaging) starting at 30 years old. The BOADICEA model was the first polygenic breast cancer risk prediction model, based on data from 2785 UK families. BOADICEA uses information from personal and family history of breast cancer, including information from breast cancer pathology, ethnicity, and *BRCA* mutations [22]. Clinical guidelines in several European countries and Switzerland recommend using BOADICEA for breast cancer risk prediction [23, 24].

However, both models have considerable limitations. BCRAT can only be used for women above 35 years old, and only takes into account history of breast cancer in first-degree relatives (mother, sisters, or daughters), without including age at diagnosis of these relatives. It does not consider family history of ovarian cancer, which may be of crucial importance for women with hereditary breast and ovarian cancer (HBOC). The BOADICEA model does not account for risk factors associated with reproductive history and hormonal exposure and has limited utility in cases with small family history. Although both models have been validated with large cohort data, their discriminatory ability, area under the ROC (receiver operating characteristics) curve, is between 0.53 and 0.64 [21, 25–28]. There is 36 to 47% chance that the BCRAT and BOADICEA model will not identify high-risk women, while some low-risk women may receive unnecessary preventive treatments. Both models make implicit assumptions that risk factors relate to cancer development in a linear way and are mostly independent from other risk factors. Thus, both models likely oversimplify complex relationships and non-linear interactions in numerous risk factors [27].

### Machine learning (ML) forecasting

ML offers an alternative approach to standard prediction modeling that may address current limitations and improve accuracy of breast cancer prediction tools [29]. ML techniques developed from earlier studies of pattern recognition and computational statistical learning. They make fewer assumptions and rely on computational algorithms and models to identify complex interactions among multiple heterogeneous risk factors. This is achieved by iteratively minimizing specific objective functions of predicted and observed outcomes [30]. ML has been used in models related to cancer prognosis and survival and produced better accuracy and reliability estimates [31–34]. To date, very few studies applied ML methods for personalized breast cancer risk prediction or compared the predictive accuracy and reliability with models commonly used in clinic practice [35]. The purpose of this study was to apply different ML techniques for forecasting individualized breast cancer risk and to compare the discriminatory accuracy of ML-based estimates against the BCRAT and BOADICEA models.

## Methods

To provide strong assessment, reliable comparison, and reproducible results, we compared ML-based estimates and estimates from BCRAT and BOADICEA model using eight synthetic simulated datasets and two actual observational datasets. In order to have fair comparisons, we used the same risk factors as BCRAT and BOADICEA models, respectively, as input for the ML algorithms in each comparison.

### Simulated datasets

We used simulated data to compare the performance between the different ML algorithms and determine the stability and validity of these predictions within each algorithm. We generated two sets of four simulated datasets (eight in total), one set consistent with the input values of BCRAT and the other consistent with the input values of the BOADICEA model. The BCRAT and BOADICEA models rely on different risk factors, which necessitated this dichotomy. For each of the two scenarios, we generated four synthetic datasets: A. simulated data with no signal (null data); B. simulated data with artificial signals; C. simulated dataset (B) adding 20% missing values; and D. simulated dataset (C) after applying multiple imputations. We randomly masked as missing 20% of values in datasets (B) to generate datasets (C), then we applied multiple imputations to datasets (C) to generate datasets (D). The cancer outcome for simulated dataset (B) for the BCRAT was simulated based on linear aggregation effects of all variables, with an artificial effect size for each variable. Variables in the null dataset (A) had no signal—these were generated with completely random values within specific ranges. In our simulation, having certain risk factors could elevate an individual’s breast cancer risk. This relative risk (signal or artificial effect size) is given according to published meta-analyses for that specific risk factor. Each individual had a baseline probability randomly assigned to them. After adding each risk factor’s attribution (RR multiplied by baseline) to baseline, we set a cutoff of the final probability to classify each sample as “healthy” or “sick”. Datasets (B) for BCRAT and BOADICEA have different input variables and data structure. For example, in data used for the BOADICEA model, each individual is imbedded into a family pedigree and have two individuals as parents. We randomly set family sizes between 3 and 80 members, and the number of generations from 1 to 5 in each family, based on our observations in the Swiss clinic-based dataset. Family members’ age and age gap between the two closest generations was set according to average age for first child birth. The pedigree (hierarchical) dataset (B) with artificial signal for the BOADICEA model was generated with R Package “pedantics,” enabling pedigree-based genetic simulation, pedigree manipulation, characterization, and viewing [36]. Multiple imputations with R package “MICE” (multivariate imputation by chained equations) [37] addressed missing data in datasets (C).

### US population-based retrospective data

We used baseline data from a prospective randomized trial conducted in Michigan (USA) including a statewide, randomly selected sample of young breast cancer survivors (YBCS) who were diagnosed with invasive breast cancer or ductal carcinoma in situ (DCIS) and their cancer-free female relatives [38, 39]. The trial recruited women diagnosed with breast cancer younger than 45 years old from the state cancer registry. The sample was stratified by race, Black versus White/Other, for adequate representation of Black YBCS. YBCS recruited cancer-free, first- and second-degree female relatives. The trial collected all information required for calculating BCRAT scores from 850 YBCS and 293 of relatives (total *n* = 1143), after excluding individuals younger than 35 years old.

### Swiss clinic-based retrospective data

The oncology department at the Geneva University Hospital (HUG) has been offering genetic evaluation and testing since 1998 to breast cancer patients and cancer-free individuals. During the genetic consultation process, information about demographic and clinical characteristics, disease history, previous genetic test results, and a detailed family pedigree are recorded with “Progeny” software [40]. Information from pathology reports, archived tumor tissue, and cancer treatment is recorded for breast cancer patients. Data from genetic consultation records and Progeny files were extracted with R packages “tm” and “gdata” [41] from 2481 families with totally 112,587 individuals. Extracted data is suitable for risk calculations with the BOADICEA model for one female member from each family. Information from 2481 women are included in this study, who are either the first female in their family to receive genetic evaluation or testing, or were a first-degree relative of a male who received genetic evaluation or testing.

### Missing values

For the US population-based dataset, there were less than 3% missing values among the variables used by the BCRAT model. For Swiss clinical datasets, there were about 13% missing values among the variables used by the BOADICEA model. Among those missing values, BRCA mutations, estrogen receptor, and progesterone receptor attributed the most (11%). Thus, missing values in BRCA mutation and hormone receptor testing were given a separate category of “unknown” in the analyses, in addition to “positive” and “negative.” This approach is also consistent with the flexibility of the BOADICEA models in handling missing information.

### Statistical analyses

Descriptive statistics, i.e., frequencies, percentages, means, and standard deviations, were computed describing sample characteristics for both categorical and continuous variables in the BRCAT and BOADICEA models and in ML approaches for *n* = 1143 US YBCS and cancer-free relatives and *n* = 2481 Swiss cancer patients and cancer-free individuals.

### BCRAT

Comparisons between ML versus BRCAT were based on performance assessment on five datasets: Simulated data A to D (*n* = 1200) and retrospective data from the U.S. population-based trial (*n* = 1143 women). The R package “brca” version 2.0 was used to calculate absolute lifetime risk of invasive breast cancer according to BCRAT algorithm for specific race/ethnic groups and age intervals for each individual in the datasets [42].

### BOADICEA model

Comparisons between ML versus the BOADICEA model were based on performance assessment on five datasets: Simulated data A to D (*n* = 2500 women) and retrospective data from HUG with 2481 females from 2481 families including 112,587 family members. Lifetime risk predictions were generated with the web-based batch processing from the BOADICEA web application. The lifetime risk for each woman was calculated using data from all the members in her family. In simulated datasets A to D, we randomly assigned a female member in each family as the index case.

### ML algorithms

We used both model-based and model-free ML techniques for predictive analytics. The model-based approaches included generalized linear models (GLM), logistic regression (LOGIT), linear discriminant analysis (LDA), Markov Chain Monte Carlo generalized linear mixed model (MCMC GLMM), and quadratic discriminant analysis (QDA) [43]. The model-free predictive analytics involved adaptive boosting (ADA), random forest (RF), and k-nearest neighbors (KNN) [43]. We selected these algorithms based on prior reports of their reliability and effectiveness in identifying, tracking, and exploiting salient features in complex, heterogeneous, and incongruent biomedical and healthcare datasets [29, 43–46]. Variables included in each comparison were listed in Table 1.

**Table 1:** Variables included in ML for comparison with BCRAT and BOADICEA

Variables list	Comparison between ML and BCRAT	Comparison between ML and BOADICEA
Age	✓	
Age at menarche	✓	
Age at first live birth	✓	
Race	✓	
Number of biopsies	✓	
Atypical hyperplasia	✓	
Number of first-degree relatives with breast cancer	✓	
Breast cancer	✓	
Family pedigree (beyond second-degree contained affected and unaffected members from both maternal and paternal side) including:		✓
Age (or age at death)		✓
Gender		✓
Deceased status		✓
Ashkenazi Jewish		✓
Ovary cancer age onset		✓
Prostate cancer age onset (male member only)		✓
Pancreatic cancer		✓
Pancreas cancer age onset		✓
Breast cancer age onset		✓
Contralateral breast cancer age onset		✓
Estrogen receptor		✓
Progesterone receptor		✓
BRCA mutation		✓

One benefit of using ML approaches was the supervised classification of breast cancer patients and cancer-free controls, where controls could outnumber patients or vice versa. We rebalanced the datasets prior to ML predictions to reduce the potential for estimate bias with the R packages “unbalanced"”(racing for unbalanced methods selection) and “SMOTE” (Synthetic Minority Over-sampling TEchnique) [47, 48]. These packages implement known ML techniques to propose a racing algorithm for adaptively selecting the most appropriate strategy for a given unbalanced task.

To ensure the reliability of ML predictions and the consistency of the forecasts, we used internal statistical n-fold cross-validation. This is an alternative strategy for validating risk estimates without a prospective dataset [49] and provides a powerful preventative measure against model overfitting [50]. Random subsampling split the entire datasets into *n* samples of equal size (*n*-folds). Each algorithm used *n* − 1 folds for training the ML algorithm and tested its accuracy with the last fold of the data in each of the n experiments. The final error estimate of the classification was obtained by averaging the n individual error estimates. We used *n* = 10 folds cross-validation with 20 repetitions in this process [51].

### Comparisons of predictive accuracy

The performance of BCRAT and the BOADICEA models were evaluated using measure of the area under the receiver operating characteristic curve (AU-ROC), while for the ML techniques the performance is presented with the mean AU-ROC from 10-fold cross validations.

### Variable importance ranking

To understand, interpret, and gain trust in the ML techniques, we identified the salient features with the highest contribution to the accuracy of these predictions by ranking them within each cross validation using training sets (*n* − 1 folds). These features were explored to ensure they are in line with both human domain knowledge and reasonable expectations. For decision tree classification methods (e.g., RF and ADA), we ranked variable importance on variable selection frequency as a decision node. For GLM, LOGIT, LDA, QDA, and MCMC GLMM algorithms, variable importance was determined by the coefficient effect size. KNN used an overall weighting of the variable within the model.

## Results

### Sample characteristics

Table 2 presents sample characteristics of the two independent observational retrospective datasets. The US population-based trial oversampled Black participants. There were more cancer cases than controls in the US sample, while the opposite was true for the Swiss sample. The average number of family members affected by breast cancer was higher in the US database, while the Swiss database included more known mutation carriers. Despite these differences, using breast cancer as an outcome grouping variable, we had sufficient number in each group even before applying a data balancing protocol.

**Table 2:** Sample characteristics of the US population-based sample (n = 1143) and the Swiss clinic-based sample (*n* = 2481)

Variables included in BCRAT and BOADICEA models and in ML algorithms	US population-based sample *n* = 1143	Swiss clinic-based sample *n* = 2481
Age (range)	50.86 ± 6.22 (35–64)	50.78 ± 12.77 (13–89)
Age at menarche (range)	12.56 ± 1.54 (8–18)	12.91 ± 1.59 (8–18)
Age at first live birth (range)	24.29 ± 5.62 (13–42)	24.13 ± 5.72 (15–48)
Number of biopsies (*n* = 847)	1.20 ± 1.21	–
Atypical hyperplasia	14 (1.65%)	–
Breast cancer	850 (74.37%)	886 (35.71%)
First-ductal carcinoma in situ (DCIS)	434 (51.06%)	50 (5.64%)
First-invasive breast cancer	404 (47.52%)	807 (91.08%)
First-breast cancer age onset (range)	40.03 ± 4.79 (26–54)	46.07 ± 10.69 (22–84)
Bilateral breast cancer	4 (0.47%)	160 (18.06%)
Estrogen receptor (ER) positive	–	618 (69.75%)
Progesterone receptor (PR) positive	–	561 (63.32%)
Pancreatic cancer	–	13 (0.52%)
Pancreatic cancer age onset (range)		55.10 ± 9.35 (36–75)
Ovarian cancer	9 (0.79%)	133 (5.36%)
Ovarian cancer age onset (range)	45.83 ± 5.00 (36–50)	56.44 ± 13.16 (21–85)
Having also breast cancer	4	20
Ethnicity (% Black)	401 (35.08%)	71 (2.86%)
Ashkenazi Jewish origin	12 (1.05%)	65 (2.29%)
Number of first-degree relatives with breast cancer	0.98 ± 1.05	0.25 ± 0.55
Breast cancer patients	0.81 ± 1.05	–
Relatives of breast cancer patients	1.49 ± 0.88	–
*BRCA1* or *BRCA2* germline mutations	32 (2.79%) 235 tested	209 (8.42%) 1052 tested

– Data not available

### Prediction accuracy

**Fig. 1:**
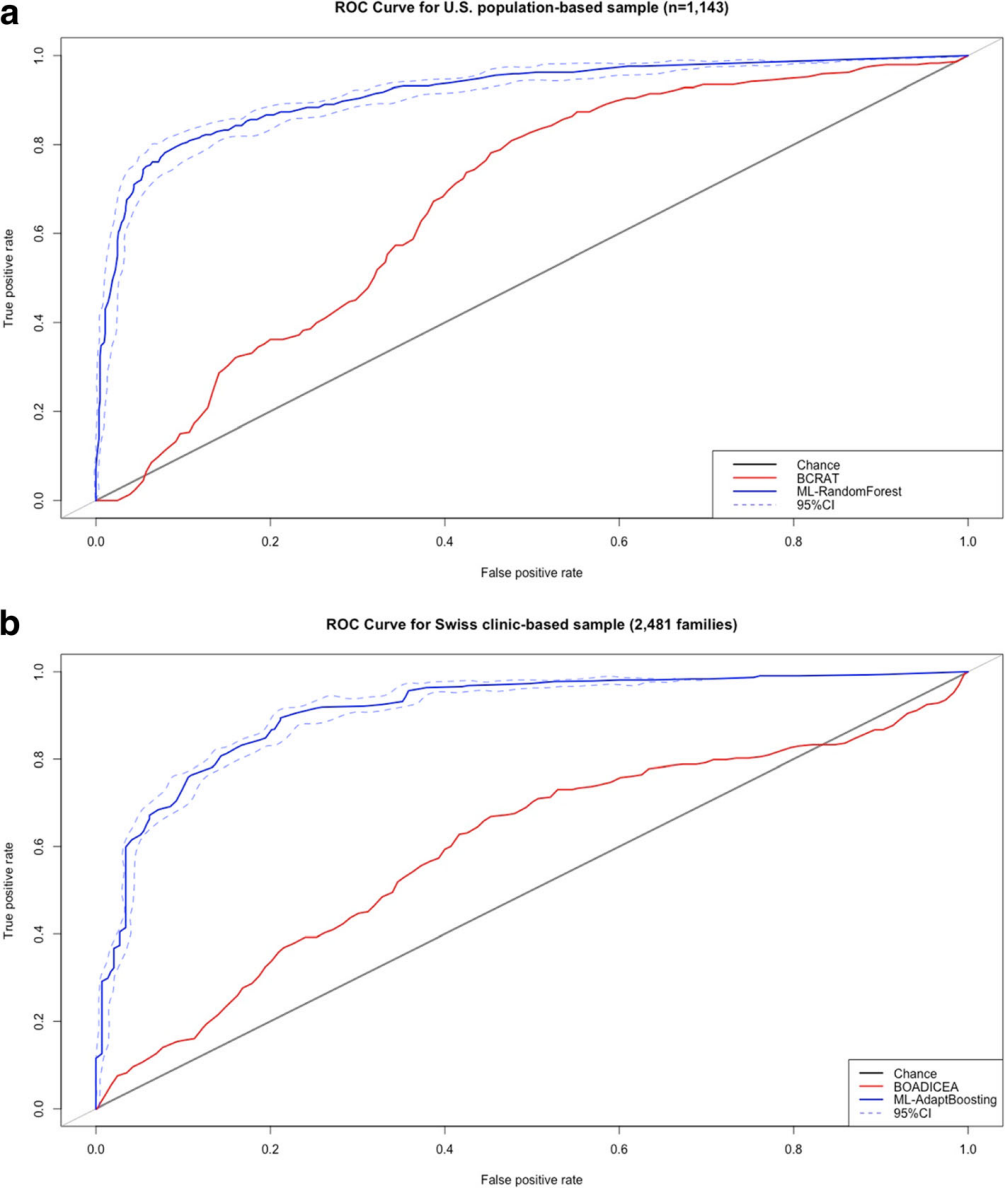
**a** The area under the receiver operating characteristic curves (AU-ROC) for BCRAT and ML-Random forest approach. **b** The area under the receiver operating characteristic curves (AU-ROC) for BOADICEA model and ML-adapt boosting approach

Tables 3 and 4 present prediction ability comparison for BCRAT and BOADICEA models and the ML techniques. In the simulated dataset A with no signal, all approaches failed to discriminate cancer cases from cancer-free controls, i.e., AU-ROCs were around 50%. In the simulated dataset B with artificial signal, most ML algorithms (except GLM) showed about 90% accuracy in prediction. The ML (except GLM) methods also maintained high accuracy (89.77–93.00%) in dataset C with 20% missing values and dataset D with multiple imputations. Using the same risk factors and similar sample sizes, the accuracy of ML techniques was superior to BCRAT and BOADICEA models in the US and Swiss observational retrospective samples. For the US population-based sample, predictive accuracy reached 88.28% using ADA and 88.89% using RF versus BCRAT AUC 62.40%. For the Swiss clinic-based sample, predictive accuracy reached 90.17% using ADA and 89.32% using MCMC GLMM versus BOADICEA AUC 59.31%. Compared to BCRAT and BOADICEA models, predictive accuracy increased by approximately 35% and 30%, respectively. In order to visualize the accuracy improvement, we generated the ROC curves in Fig. 1a, b from predictions of BCRAT and BOADICEA models and one ML approaches performed best.

**Table 3:** Performance AU-ROC curve of BCRAT and ML algorithms (with standard deviation) predicting breast cancer lifetime risk from simulated datasets (*n* = 1200) and the US population-based sample (*n* = 1143)

Dataset	BCRAT	ML: random forest	ML: Logistic Regression	ML: adapt boosting	ML: Linear Model	ML: K-nearest neighbors	ML: linear discriminant	ML: quadratic discriminant	ML: MCMC GLMM
A.Sim_no_signal	0.5333	0.5016 (0.0231)	0.5133 (0.0271)	0.5067 (0.0307)	0.5015 (0.0220)	0.5054 (0.0211)	0.5158 (0.0276)	0.5133 (0.0323)	0.5090 (0.0210)
B.Sim_atifical_signal	0.5261	0.9308 (0.0171)	0.9417 (0.0103)	0.9292 (0.0095)	0.7859 (0.0197)	0.9125 (0.0109)	0.9312 (0.0154)	0.9188 (0.0111)	0.9329 (0.0087)
C. Sim_ atifical_signal + 20% missing	0.5068	0.9275 (0.0179)	0.9217 (0.0259)	0.9258 (0.0113)	0.7807 (0.0227)	0.9012 (0.0120)	0.9213 (0.0202)	0.9104 (0.0237)	0.9191 (0.0210)
D. Sim_ atifical_signal + 20% missing + imputation	0.5035	0.9167 (0.0184)	0.9300 (0.0111)	0.9213 (0.0119)	0.7824 (0.0200)	0.9058 (0.0117)	0.9275 (0.0148)	0.9121 (0.0081)	0.9232 (0.0099)
US population-based sample	0.6240	0.8889 (0.0201)	0.7192 (0.0314)	0.8828 (0.0229)	0.6813 (0.0378)	0.8089 (0.0217)	0.8692 (0.0284)	0.8675 (0.0241)	0.8234 (0.0189)

**Table 4:** Performance AU-ROC curve of the BOADICEA model and ML algorithms (with standard deviation) predicting breast cancer lifetime risk from simulated datasets (n = 2500) and Swiss clinic-based sample (*n* = 112,587 women from 2481 families)

Dataset	BOADICEA model	ML: random forest	ML: logistic regression	ML: adapt boosting	ML: linear model	ML: K-nearest neighbors	ML: linear discriminant	ML: quadratic discriminant	ML: MCMC GLMM
A.Sim_no_signal	0.5103	0.5020 (0.0197)	0.5093 (0.0210)	0.5029 (0.0177)	0.5151 (0.0190)	0.5254 (0.0199)	0.5094 (0.0241)	0.5002 (0.0216)	0.5075 (0.0201)
B.Sim_ atifical_signal	0.5392	0.9101 (0.0148)	0.9233 (0.0172)	0.9321 (0.0122)	0.6659 (0.0164)	0.9301 (0.0159)	0.9109 (0.0187)	0.9244 (0.0166)	0.9219 (0.0151)
C.Sim_ atifical_signal + 20% missing	0.5022	0.8977 (0.0183)	0.9100 (0.0293)	0.9291 (0.0156)	0.6407 (0.0257)	0.9232 (0.0180)	0.8982 (0.0276)	0.9209 (0.0297)	0.9088 (0.0219)
D.Sim_ atifical_signal + 20% missing +imputation	0.5115	0.9028 (0.0127)	0.9203 (0.0157)	0.9299 (0.0110)	0.6463 (0.0147)	0.9276 (0.0140)	0.9035 (0.0159)	0.9220 (0.0141)	0.9154 (0.0137)
Swiss clinic-based sample	0.5931	0.8535 (0.0214)	0.8271 (0.0189)	0.9017 (0.0162)	0.6921 (0.0202)	0.8377 (0.0156)	0.7899 (0.0188)	0.8369 (0.0192)	0.8932 (0.0149)

### ML variable importance rankings

Tables 5 and 6 present the most influential variables in different ML algorithms and the relative rank of the top five variables in decreasing order. In the US population-based sample, three of the risk factors included in BCRAT (number of biopsies, age, and number of first-degree relatives with breast cancer) were the top-ranked risk factors for almost all ML algorithms, except for LDA. Four ML algorithms (RF, ADA, KNN, and MCMC GLMM) identified number of biopsies as the most important risk factor for discriminatory accuracy (Table 5). For the Swiss clinic-based sample, two of the risk factors included in the BOADICEA model (age, family history) were the top-ranked risk factors for all ML algorithms, except for KNN and QDA (Table 6).

**Table 5:** Top five important risk factors in descending order for different ML algorithms based on the US population-based training samples in 10-fold internal statistical cross-validations

ML: random forest	ML: logistic regression	ML: adapt boosting	ML: linear model	ML: K-nearest neighbors	ML: linear discriminant	ML: quadratic discriminant	ML: MCMC GLMM
Number of biopsies	Number of first-degree relatives with breast cancer	Number of biopsies	Age	Number of biopsies	Age	Number of first-degree relatives with breast cancer	Number of biopsies
Age	Age	Age	Number of biopsies	Number of first-degree relatives with breast cancer	Number of biopsies	Number of biopsies	Age
Number of first-degree relatives with breast cancer	Number of biopsies	Number of first-degree relatives with breast cancer	Number of first-degree relatives with breast cancer	Age	Ethnicity	Age	Number of first-degree relatives with breast cancer
Age at menarche	Ethnicity	Age at menarche	Age at menarche	Ethnicity	Number of first-degree relatives with breast cancer	Ethnicity	Age at first live birth
Ethnicity	Age at first live birth	Ethnicity	Age at first live birth	Age at first live birth	Age at first live birth	Age at menarche	Age at menarche

**Table 6:** Top five important risk factors in descending order for different ML algorithms based on the Swiss clinical-based training samples in 10-fold internal statistical cross-validations

ML: random forest	ML: logistic regression	ML: adapt boosting	ML: linear model	ML: K-nearest neighbors	ML: linear discriminant	ML: quadratic discriminant	ML: MCMC GLMM
Breast cancer age onset	Age	Breast cancer age onset	Age	Family history	Age	Breast cancer age onset	Breast cancer age onset
Age	Breast cancer age onset	Age	Breast cancer age onset	Mutation	Breast cancer age onset	Mutation	Age
Mutation	Ashkenazi Jewish origin	Mutation	Ashkenazi Jewish origin	Age	Mutation	Age	Mutation
Ashkenazi Jewish origin	Ovarian cancer age onset	Ashkenazi Jewish origin	Mutation	Ashkenazi Jewish origin	Ashkenazi Jewish origin	Ashkenazi Jewish origin	Ovarian cancer age onset
Ovarian cancer age onset	Mutation	Ovarian cancer age onset	Ovarian cancer age onset	Ovarian cancer age onset	Ovarian cancer age onset	Ovarian cancer age onset	Ashkenazi Jewish origin

## Discussion

We examined whether using ML algorithms could improve breast cancer predictive accuracy compared to the BCRAT and BOADICEA models. We computed the predictive accuracy of these two models and eight different ML algorithms using datasets with artificial signals (datasets B to D) and two observational retrospective datasets from two different countries and different target samples (population-based versus clinic-based). Compared to BCRAT and the BOADICEA models, most ML techniques we tested were superior at distinguishing cancer cases from cancer-free controls. ML algorithms improved significantly the predictive accuracy of both models from less than 0.65 to about 0.90, especially when tested with real samples. ML algorithms that produced the best accuracy were ADA followed by RF using variables of BCRAT, and the MCMC GLMM using variables of the BOADICEA model. The increased predictive accuracy observed with ML algorithms was not due to additional input variables, since we used exactly the same risk factors as the BCRAT and the BOADICEA models. Rather, this was due to inherently better predictive ability of ML algorithms. With supervised learning approaches, the artificial or natural complexities of each dataset were restored and adhered to different algorithms with high accuracy. When the datasets were intentionally perturbed by introducing missing values or performing multiple imputations, the prediction performance of the ML algorithms remained stable.

Using different simulated datasets allow us to control the input and assess the case-classification/prediction results relative to “ground truth.” We simulated dataset (A) as a “null” reference case-study. This helps us identify false-positive predictions, because when no signal exists in the dataset, all approaches should fail to classify the samples. In simulated datasets (B), (C), and (D), we created the artificial signals within the datasets to strongly correlate with the outcome (breast cancer yes/no). This approach allows us to test whether the machine learning algorithms we used can detect these artificial signals and provide valid and stable predictions, even when there are missing values. This helps us identify false-negative predictions.

In the simulated datasets, we assigned estimations (e.g., coefficient or weight) to each risk factor based on published epidemiological data. Unfortunately, there is no available information about the underlying estimation of each risk factor used in the BCRAT and BOADICEA models. The only available information is that these estimations are derived from large cohort studies over time. Therefore, it is possible that the estimations in the simulated datasets are different from the estimations used by the BCRAT and BOADICEA models, which may explain the underperformance of the later models to predict the class in the simulated datasets. Moreover, the simulated datasets have oversimplified artificial signals, which make it relatively easier for the more general approaches of machine learning to pick up a signal and identify features in the controlled simulated data than in real datasets. Thus, the machine learning-based algorithms showed opposite trends on simulated data compared to the model-based methods. Finally, the simulated datasets were not used for a comparison between the machine learning algorithms and the BCRAT or the BOADICEA model. The main purpose of using simulated datasets was to compare predictions between different machine learning algorithms and the stability within each machine learning method.

Ranking importance of variables in each model was consistent with our expectations. Biopsy testing indicated suspicious cell abnormality. Number of first-degree relatives affected with breast cancer as well as cancer age onset in a family pedigree can partially reflect the common environmental exposures, inherited information, and lifestyles. We observed variations and similarities in the importance of risk factors depending on the core algorithms in each ML approach and variable types. ADA and RF were both based on decision trees and resembled closely in variables and ranking. QDA placed more importance on categorical variables, e.g., number of first-degree relatives with breast cancer, while LDA placed more importance on continuous variable, e.g., age in both comparisons. This finding has implications for future research aiming to develop a new breast cancer risk prediction model, incorporating established and newly evaluated risk factors.

As firm supporters of “open-science,” we have packaged, documented, and distributed the complete end-to-end R-protocol used to generate the synthetic data and perform all data analytics reported in this manuscript. We have shared the protocol via GitHub (https://github.com/SOCR/ML_BCP/).

### Strengths and limitations

The inclusion-exclusion selection criteria of the US and the Swiss datasets may have influenced the association between observed variance and outcomes. In the US population-based sample, YBCS had fewer affected relatives than their cancer-free relatives. Thus, number of affected relatives was detected as an important variable but without external validity in interpretation. Interpretability of the function modeled by ML algorithms is only partially limited by the “black-box” nature of ML algorithms in our study because we included a limited number of well-established breast cancer risk factors. However, the inherent complexity of how risk factors interact with each other, their independent effect on the outcome, and how effect sizes are determined within each ML algorithm is not known.

Significant strengths of the study include the novelty of the approach, i.e., applying ML algorithms in individual breast cancer risk prediction and comparing predictive accuracy with existing models. The improvement achieved with ML algorithms in accurate classification of women with and without breast cancer compared to the state-of-the-art model-based approaches was striking. We demonstrated a range of ML algorithms with cross-validations, which is lacking in other applications of ML for cancer prognosis [32]. Different ML algorithms for feature selection and classification showed great adaptability and discriminatory accuracy in our study by handling multidimensional and heterogeneous data. Ranking variable importance may inform algorithm selection with diverse predictive risk factors for future development of new risk prediction models.

## Conclusions

Predictive models are essential in personalized medicine because they contribute to early identification of high-risk individuals based on known epidemiological and clinical risk factors. Accurate breast cancer risk estimates can inform clinical care and risk management across the breast cancer continuum, e.g., behavioral changes, chemoprevention, personalized screening, and risk-stratified follow-up care. Available risk prediction models have an overall accuracy less than 0.65. ML approaches offer the exciting prospect of achieving improved and more precise risk estimates. This is the first step in developing new risk prediction approaches and further explores diverse risk factors. ML algorithms are not limited to a specific number of risk factors but have the flexibility to change or incorporate additional ones. The improvement in predictive accuracy achieved in this study should be further explored and duplicated with prospective databases and additional risk factors, e.g., mammographic density, risk factors in IBIS Breast Cancer Risk Evaluation Tool, and polygenic genetic scores. Improvements in computational capacity and data management in healthcare systems can be followed by opportunities to exploit ML to enhance risk prediction of disease and survival prognosis in clinical practice [52].

## Abbreviations

ADA: Adaptive boosting analysis
AU-ROC curve: Area under the receiver operating characteristics curve
BCRAT: Breast Cancer Risk Assessment Tool
BOADICEA: Breast and Ovarian Analysis of Disease Incidence and Carrier Estimation Algorithm
GLM: Generalized linear model
HBOC: Hereditary breast and ovarian cancer
HUG: Geneva University Hospital
IBIS: Breast Cancer Risk Evaluation Tool
KNN: K-nearest neighbors
LDA: Linear discriminant analysis
LOGIT: Logistic regression
MCMC GLMM: Markov chain Monte Carlo generalized linear mixed model
ML: Machine learning
MRI: Magnetic resonance imaging
QDA: Quadratic discriminant analysis
RF: Random forest
YBCS: Young breast cancer survivor

## References

[1] Nelson HD, Tyne K, Naik A, Bougatsos C, Chan BK, Humphrey L (2009). Screening for breast cancer: an update for the U.S. Preventive Services Task Force. Ann Intern Med.

[2] Arie S. Switzerland debates dismantling its breast cancer screening programme. BMJ. 2014;348. https://www.bmj.com/content/348/bmj.g1625.ful. 10.1136/bmj.g1625 24550244

[3] Christine Bouchardy PP, Lorez M, Clough-Gorr K, Bordoni A, the NICER Working Group. Trends in Breast Cancer Survival in Switzerland. NICER. Zurich: Schweizer Krebsbulletin(Nr. 4/2011); 2011.

[4] Mainiero MB, Moy L, Baron P, Didwania AD, diFlorio RM, Green ED (2017). ACR Appropriateness Criteria((R)) breast cancer screening. J Am Coll Radiol.

[5] Qin X, Tangka FK, Guy GP, Howard DH (2017). Mammography rates after the 2009 revision to the United States Preventive Services Task Force breast cancer screening recommendation. Cancer Causes Control.

[6] Sardanelli F, Aase HS, Alvarez M, Azavedo E, Baarslag HJ, Balleyguier C (2017). Position paper on screening for breast cancer by the European Society of Breast Imaging (EUSOBI) and 30 national breast radiology bodies from Austria, Belgium, Bosnia and Herzegovina, Bulgaria, Croatia, Czech Republic, Denmark, Estonia, Finland, France, Germany, Greece, Hungary, Iceland, Ireland, Italy, Israel, Lithuania, Moldova, The Netherlands, Norway, Poland, Portugal, Romania, Serbia, Slovakia, Spain, Sweden, Switzerland and Turkey. Eur Radiol.

[7] King MC, Levy-Lahad E, Lahad A (2014). Population-based screening for BRCA1 and BRCA2: 2014 Lasker Award. Jama..

[8] Azim HA, Partridge AH (2014). Biology of breast cancer in young women. Breast Cancer Res..

[9] Rosenberg SM, Newman LA, Partridge AH (2015). Breast cancer in young women: rare disease or public health problem?. JAMA Oncol.

[10] Autier P, Boniol M (2018). Mammography screening: a major issue in medicine. Eur J Cancer.

[11] van Ravesteyn NT, Miglioretti DL, Stout NK, Lee SJ, Schechter CB, Buist DS (2012). Tipping the balance of benefits and harms to favor screening mammography starting at age 40 years: a comparative modeling study of risk. Ann Intern Med.

[12] Eccles SA, Aboagye EO, Ali S, Anderson AS, Armes J, Berditchevski F (2013). Critical research gaps and translational priorities for the successful prevention and treatment of breast cancer. Breast Cancer Res.

[13] Maas P, Barrdahl M, Joshi AD, Auer PL, Gaudet MM, Milne RL (2016). Breast cancer risk from modifiable and nonmodifiable risk factors among White women in the United States. JAMA Oncol..

[14] Mandelblatt JS, Cronin KA, Bailey S, Berry DA, de Koning HJ, Draisma G (2009). Effects of mammography screening under different screening schedules: model estimates of potential benefits and harms. Ann Intern Med.

[15] Pashayan N, Duffy SW, Chowdhury S, Dent T, Burton H, Neal DE (2011). Polygenic susceptibility to prostate and breast cancer: implications for personalised screening. Br J Cancer.

[16] Schousboe JT, Kerlikowske K, Loh A, Cummings SR (2011). Personalizing mammography by breast density and other risk factors for breast cancer: analysis of health benefits and cost-effectiveness. Ann Intern Med.

[17] Vilaprinyo E, Forne C, Carles M, Sala M, Pla R, Castells X (2014). Cost-effectiveness and harm-benefit analyses of risk-based screening strategies for breast cancer. PLoS One.

[18] Visvanathan K, Hurley P, Bantug E, Brown P, Col NF, Cuzick J (2013). Use of pharmacologic interventions for breast cancer risk reduction: American Society of Clinical Oncology clinical practice guideline. J Clin Oncol.

[19] Moyer VA (2013). Medications to decrease the risk for breast cancer in women: recommendations from the U.S. Preventive Services Task Force recommendation statement. Ann Intern Med.

[20] Gail MH, Brinton LA, Byar DP, Corle DK, Green SB, Schairer C (1989). Projecting individualized probabilities of developing breast cancer for white females who are being examined annually. J Natl Cancer Inst.

[21] Wang X, Huang Y, Li L, Dai H, Song F, Chen K (2018). Assessment of performance of the Gail model for predicting breast cancer risk: a systematic review and meta-analysis with trial sequential analysis. Breast Cancer Res.

[22] Antoniou AC, Cunningham AP, Peto J, Evans DG, Lalloo F, Narod SA (2008). The BOADICEA model of genetic susceptibility to breast and ovarian cancers: updates and extensions. Br J Cancer.

[23] Usher-Smith J, Emery J, Hamilton W, Griffin SJ, Walter FM (2015). Risk prediction tools for cancer in primary care. Br J Cancer.

[24] Gagnon JLE (2016). The Clinical Advisory Committee on Breast Cancer Screening and Prevention, et al. Recommendations on breast cancer screening and prevention in the context of implementing risk stratification: impending changes to current policies. Curr Oncol.

[25] Amir E, Evans DG, Shenton A, Lalloo F, Moran A, Boggis C (2003). Evaluation of breast cancer risk assessment packages in the family history evaluation and screening programme. J Med Genet.

[26] Brentnall AR, Harkness EF, Astley SM, Donnelly LS, Stavrinos P, Sampson S (2015). Mammographic density adds accuracy to both the Tyrer-Cuzick and Gail breast cancer risk models in a prospective UK screening cohort. Breast Cancer Res.

[27] Meads C, Ahmed I, Riley RD (2012). A systematic review of breast cancer incidence risk prediction models with meta-analysis of their performance. Breast Cancer Res Treat.

[28] Tice JA, Cummings SR, Smith-Bindman R, Ichikawa L, Barlow WE, Kerlikowske K (2008). Using clinical factors and mammographic breast density to estimate breast cancer risk: development and validation of a new predictive model. Ann Intern Med.

[29] Obermeyer Z, Emanuel EJ (2016). Predicting the future - big data, machine learning, and clinical medicine. N Engl J Med.

[30] Dreiseitl S, Ohno-Machado L (2002). Logistic regression and artificial neural network classification models: a methodology review. J Biomed Inform.

[31] Chen HC, Kodell RL, Cheng KF, Chen JJ (2012). Assessment of performance of survival prediction models for cancer prognosis. BMC Med Res Methodol.

[32] Kourou K, Exarchos TP, Exarchos KP, Karamouzis MV, Fotiadis DI (2015). Machine learning applications in cancer prognosis and prediction. Comput Struct Biotechnol J.

[33] Reinbolt RE, Sonis S, Timmers CD, Fernandez-Martinez JL, Cernea A, de Andres-Galiana EJ (2018). Genomic risk prediction of aromatase inhibitor-related arthralgia in patients with breast cancer using a novel machine-learning algorithm. Cancer Med.

[34] Vanneschi L, Farinaccio A, Mauri G, Antoniotti M, Provero P, Giacobini M (2011). A comparison of machine learning techniques for survival prediction in breast cancer. BioData Min.

[35] Heidari M, Khuzani AZ, Hollingsworth AB, Danala G, Mirniaharikandehei S, Qiu Y (2018). Prediction of breast cancer risk using a machine learning approach embedded with a locality preserving projection algorithm. Phys Med Biol.

[36] Morrissey M (2018). Pedantics: functions to facilitate power and sensitivity analyses for genetic studies of natural populations.

[37] van Buuren S, Groothuis-Oudshoorn K, Robitzsch A, Vink G, Doove L, Jolani S, Schouten R, Gaffert P, Meinfelder F, Gray B (2017). MICE: multivariate imputation by chained equations.

[38] Katapodi MC, Northouse LL, Schafenacker AM, Duquette D, Duffy SA, Ronis DL (2013). Using a state cancer registry to recruit young breast cancer survivors and high-risk relatives: protocol of a randomized trial testing the efficacy of a targeted versus a tailored intervention to increase breast cancer screening. BMC Cancer.

[39] Katapodi MC, Duquette D, Yang JJ, Mendelsohn-Victor K, Anderson B, Nikolaidis C (2017). Recruiting families at risk for hereditary breast and ovarian cancer from a statewide cancer registry: a methodological study. Cancer Causes Control..

[40] Progeny 9, Version March 2018. Family data and pedigree information was stored and manipulated using the genetic data management system (Progeny CLINICAL Version N) from Progeny Software (Progeny Software LLC, Delray Beach, FL www.progenygenetics.com).

[41] Team RC. R: a language and environment for statistical computing. Vienna: R Foundation for Statistical Computing; 2017.

[42] Zhang F (2018). Breast cancer risk assessment. 2.0 ed.

[43] Dinov Ivo D. (2018). Data Science and Predictive Analytics.

[44] Murdoch TB, Detsky AS (2013). The inevitable application of big data to health care. JAMA..

[45] Toga AW, Dinov ID (2015). Sharing big biomedical data. J Big Data.

[46] Dinov ID, Heavner B, Tang M, Glusman G, Chard K, Darcy M (2016). Predictive big data analytics: a study of Parkinson’s disease using large, complex, heterogeneous, incongruent, multi-source and incomplete observations. PLoS One.

[47] Pozzolo AD, Caelen O, Bontempi G (2015). unbalanced: racing for unbalanced methods selection.

[48] Chawla N, Bowyer K, Hall L, Kegelmeyer W (2002). SMOTE: synthetic minority over-sampling technique. J Art Intell Res.

[49] Kohavi R (1995). A study of cross-validation and bootstrap for accuracy estimation and model selection.

[50] Ng AY. Preventing “Overfitting” of Cross-Validation Data. In: Proceedings of the Fourteenth International Conference on Machine Learning, vol. 657119. Burlington: Morgan Kaufmann Publishers Inc; 1997. p. 245–53.

[51] Strimme K (2015). Package ‘crossval’. Contains generic functions for performing cross validation and for computing diagnostic errors.

[52] Hickey KT, Katapodi MC, Coleman B, Reuter-Rice K, Starkweather AR (2017). Improving utilization of the family history in the electronic health record. J Nurs Scholarsh.

